# Research on Segmentation Technology in Lung Cancer Radiotherapy Based on Deep Learning

**DOI:** 10.2174/1573405619666230123104243

**Published:** 2023-05-31

**Authors:** Jun Huang, Tao Liu, Beibei Qian, Zhibo Chen, Ya Wang

**Affiliations:** 1 School of Computer and Information Engineering, Fuyang Normal University, Fuyang Anhuis 236037, China

**Keywords:** Lung cancer, deep learning, image segmentation, organs at risk, lung tumors, radiation therapy

## Abstract

**Background:**

Lung cancer has the highest mortality rate among cancers. Radiation therapy (RT) is one of the most effective therapies for lung cancer. The correct segmentation of lung tumors (LTs) and organs at risk (OARs) is the cornerstone of successful RT.

**Methods:**

We searched four databases for relevant material published in the last 10 years: Web of Science, PubMed, Science Direct, and Google Scholar. The advancement of deep learning-based segmentation technology for lung cancer radiotherapy (DSLC) research was examined from the perspectives of LTs and OARs.

**Results:**

In this paper, Most of the dice similarity coefficient (DSC) values of LT segmentation in the surveyed literature were above 0.7, whereas the DSC indicators of OAR segmentation were all over 0.8.

**Conclusion:**

The contribution of this review is to summarize DSLC research methods and the issues that DSLC faces are discussed, as well as possible viable solutions. The purpose of this review is to encourage collaboration among experts in lung cancer radiotherapy and DL and to promote more research into the use of DL in lung cancer radiotherapy.

## 1 INTRODUCTION

### 1.1 Motivation

Lung cancer is the deadliest cancer in the world [1, 2]. Fig. (**1**) depicts WHO's global cancer data from 2020, which reveal that there were around 1.8 million fatal cases, the highest mortality rate of all cancer categories [3].

In recent years, radiation therapy (RT) has made great technological progress and has played an irreplaceable role in the treatment of lung cancer [4-8]; more than 50% of patients with malignant tumors need to receive RT [9]. The fundamental purpose of RT is to maximize the radiation dose to the target area to kill tumor cells while reducing or avoiding unnecessary radiation to the surrounding organs at risk (OARs). Therefore, the gross tumor volume (GTV), clinical target volume (CTV), and OARs should be accurately segmented in RT planning [10]. At present, automatic segmentation technology based on the atlas is more mature [11-13]; however, the biggest disadvantage of this technology is that it relies heavily on similarities between images. In recent years, several automatic segmentation techniques based on deep learning have been proposed [14-17]. Deep learning (DL) has been widely used in oncology, radiology, and other medical fields to better assist doctors with disease prediction and diagnosis [17-24]. DL in lung cancer radiotherapy segmentation can help doctors not only get more accurate and effective segmentation results [25-31], but also reduce the workload of manually segmenting patient images, allowing them to spend more time on optimizing radiotherapy plans.

### 1.2 Contribution

In this paper, we investigate the application of DL to radiotherapy in lung cancer, conduct an extensive survey of OAR and lung tumor (LT) segmentation, and compare different segmentation methods based on DL. Section 2 introduces the research strategy of the paper and some commonly used lung cancer datasets and compares this study with related work. Section 3 describes the basic knowledge and evaluation indicators of DL, and focuses on the two clinical application points of LT and OAR segmentation in the process of lung cancer radiotherapy. Section 4 discusses current challenges and possible solutions. Finally, the paper is concluded in Section 5. We investigated many pieces of literature and found that there are few reviews on deep learning-based segmentation technology for lung cancer radiotherapy (DSLC). This paper aims to present the latest developments in DSLC for researchers and provide readers with a convenient reference.

## 2 LITERATURE SEARCH

A large amount of literature was read during the research for this paper. This section explains the approach and selection criteria for conducting a literature search in detail. There is also a summary of commonly used lung datasets.

### 2.1 Search Policies and Criteria

We retrieved relevant literature from the last ten years using four databases: Web of Science, PubMed, Science Direct, and Google Scholar. The following keyword combinations were employed in the search process: “Lung cancer,” “Radiotherapy for lung cancer,” “Lung segmentation,” “Lung tumor segmentation,” “Artificial intelligence.” The queried results were imported into Endnote for deduplication [32], and there were 2183 literature items obtained after filtering. In this section, we used Endnote to analyze these papers.

Fig. (**2**) shows a chart of the relevant literature over the last 10 years. Fig. (**2a**) shows the publication trend: the number of articles published in this direction increased by 148% in the past three years, but the overall number of articles published remained low. Fig. (**2b**) is a keyword analysis diagram of papers in related areas in the past 10 years, among which DL accounts for a large proportion of the word cloud. To sum up, the data show that DSLC is a hot research topic that has emerged in recent years.

### 2.2 Literature Survey

Table **1** compares five review articles on OAR and LT segmentation and detection in the past five years. This survey mainly analyzed the deficiencies of the literature in terms of coverage, data indicators, and research trends.

The results in Table **1** indicate some limitations in the existing reviews. First, there is a lack of detailed reviews explaining the limitations of other studies and the motivation for their own research; second, there is a lack of analysis of research trends; third, there is a lack of evaluation of relevant research work, metric details, and dataset details; and fourth, there is a lack of discussion of current research challenges and possible solutions. We conducted a detailed DSLC survey in an attempt to fill the gaps in the existing literature.

### 2.3 Common Datasets for Lung Tumors

Some publicly available datasets are frequently used in the diagnosis and treatment of lung cancer using deep neural networks, as shown in (Table **2**).

Among the lung cancer datasets listed in Table **2**, the LIDC-IDRI dataset provides an authoritative and open standard for research on lung nodules [55, 56], and the details of other lung cancer-related datasets are also summarized in the table.

## 3 DEEP LEARNING AUTOMATIC SEGMENTATION TECHNOLOGY

### 3.1 Introduction to Deep Learning

DL has been widely used in image analysis in pathology [57-59]. The current popular DL algorithm includes a convolutional neural network (CNN) [60] and generative adversarial network (GAN) [61]; the latter has the characteristics of unsupervised learning [62]. Some scholars integrate GAN and CNN for medical image segmentation. Based on the wider application of CNN, in this paper, we focus on the application of CNN in DSLC. CNN contains convolutional, pooling, and fully connected layers. The role of the convolutional layer is to use the convolution kernel to extract features from the input image. The role of the pooling layer is to reduce the resolution of the feature map and the consumption of memory. The role of the fully connected layer is to classify and output the extracted features. The structure diagram of CNN is shown in Fig. (**3**).

Commonly used basic CNNs are VGG [63] and ResNet [64]. VGG is a network model with a simple structure and strong generalization ability. VGG increases the receptive field by stacking small convolution kernels. ResNet is based on the concept of using shortcut connections to solve the problem of deep network degradation so that thousands of layers of convolutional networks can converge.

In addition to the basic convolutional network, there are two commonly used segmentation neural networks, FCN [65] and U-Net [66]. FCN uses a skip connection structure to fuse the shallow appearance information and deep semantic information of the feature map to segment images more accurately. U-Net has a better processing effect for medical image data with a small amount of data, large image size, blurred boundaries, and multi-modal imagery, and has become the baseline for most medical image semantic segmentation tasks. In addition, the derived Attention U-Net [67] further improves the performance of image segmentation.

### 3.2 Common Evaluation Indicators

Table **3** lists the metrics commonly used in experiments; among them, the dice similarity coefficient (DSC) is a simple and useful statistical validation metric that can be applied to study the accuracy of image segmentation [68].

### 3.3 LT and OAR Segmentation for Lung Cancer

**Fig. (4):**
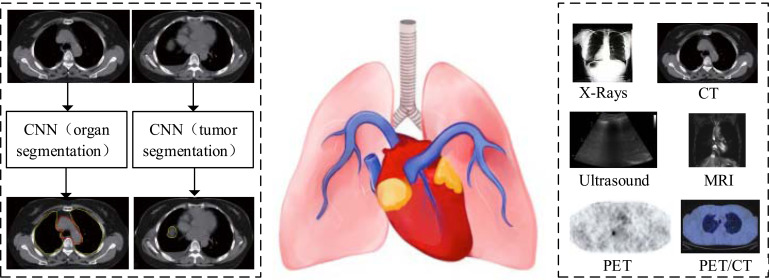
Segmentation of lung tumors (LTs) and organs at risk (OARs) for lung cancer.

Patients with advanced lung cancer have a five-year survival rate of less than 15%, but survival rates after treatment for early-stage lung cancer can range from 40 to 70% [75]. As a result, early detection and treatment are critical to increasing the cure rate [76]. The primary treatment method for lung cancer is RT. In clinical practice, precise irradiation of tumor target areas and protection of OARs are critical factors for RT success, and DSLC plays an important role in these tasks. This section discusses and compares DSLC-related work from two perspectives: LT segmentation and OAR segmentation (Fig. **4**).

#### 3.3.1 Lung Tumor Segmentation

In the diagnosis of clinical lung tumors (LTs), it is often necessary to process images of different modalities, such as X-ray, computed tomography (CT), ultrasound, magnetic resonance imaging (MRI), positron emission tomography (PET), and positron emission computed tomography (PET-CT), as shown in Fig. (**4**).

Zhang *et al*. [77] developed an improved ResNet for segmenting of non-small-cell lung tumors on CT images, combining shallow and deep semantic features to produce dense pixel output. In 2020, Pang *et al*. [78] proposed CTumorGAN, a unified end-to-end adversarial learning framework, for the prediction of CT images using multi-level supervision of different modules to deal with problems such as class imbalance, small tumors, and label noise, with a DSC coefficient of 71.08%. With a success rate of 99.92%, the method improves the model's generalization ability for different objective functions and achieves a stable tumor segmentation scheme with a low error rate. Jiang J. *et al*. [79] developed a cross-modal (MR-CT) depth learning segmentation method, which enhances training data by converting manually segmented CT images into pseudo-MR images.

MRI provides high resolution for soft tissue, allowing a better view of tumors and adjacent normal tissues. Wang *et al*. [29] presented A-Net, a new patient-specific adaptive convolutional neural network that uses MRI imags and GTV annotation to train the network model; its DSC index and precision are 0.82 0.10 and 0.81 0.08, respectively Jiang *et al*. [80] developed a cross-modality induced distillation method for cone-beam CT (CBCT) images. The idea is to use MRI to guide the training of the CBCT segmentation network.

The advantage of PET is that it can accurately locate small tumors and distinguish benign and malignant tumors early. Leung *et al*. [81] proposed mU-Net for segmenting of PET images, which is designed to help address the challenge of a lack of clinical training data with known ground-truth tumor boundaries in PET.

PET-CT combines the high sensitivity of PET images with the anatomical information of CT images and overcomes the difficulties of blurred image boundaries, low contrast, and complex backgrounds. Zhao *et al*. [74] proposed a multimodal segmentation method based on 3D full convolution neural network, which can extract the characteristic information of PET and CT simultaneously for tumor segmentation, and has strong robustness. In 2020, Li *et al*. [82] integrated CT tumor probability maps and PET images into a recognition model, which could accurately identify the input images. In 2021, Lei *et al*. [83] proposed a recurrent fusion network (RFN) for automatic PET-CT tumor segmentation that can complementarily fuse the intermediate segmentation results to obtain multi-modal image features, which improves the convergence speed. Fu *et al*. [84] proposed a multi-modal spatial attention network module (MSAM).

In addition, Bi *et al*. [85] established a deep expansion residual network based on ResNet-101, which is used to automatically sketch the CTV of lung cancer patients undergoing radiotherapy after surgery. The experimental results show that, compared with manual contour, the effect of deep learning assisted sketching is better, and 35% of the time is saved than before. Jemaa *et al*. [86] proposed an end-to-end method to quickly identify and segment tumors by combining 2D and 3D convolutional networks, which can adapt to an extreme imbalance between healthy tissue volumes and heterogeneity of input images. Jiang *et al*. [87] developed two multiresolution residual connection networks, combined the features and functional levels of multiple image resolutions, and detected and segmented lung tumors through residual connection. After evaluation, it can accurately segment the volume of lung tumors.

Table **4** lists the lung tumor segmentation work in detail. Fig. (**5**) shows the DSC accuracy of lung tumor segmentation in the related literature, where the abscissa represents the reference numbers in Table **4** and the ordinate represents the DSC values, which are mostly above 0.7 [88].

#### 3.3.2 Organ-at-Risk Segmentation

Because RT can affect organs outside the target area, radiation oncologists must accurately segment OARs to reduce the probability of normal tissue complications after RT. DL segmentation models can now automatically segment OARs based on trial and error. This section discusses various methods for solving the difficult problem of automatic OAR segmentation, such as experimenting with different network architectures, introducing loss functions, and combining supervised and unsupervised learning methods, which will be discussed in detail below. Zhu *et al*. [89] improved the deep learning split network based on U-Net, which can split many kinds of OARs in the lung. Among them, the DSC index for segmenting the lung is the highest, reaching 95%. Feng *et al*. [73] proposed a based 3D U-Net model to automatically segment five sternal OARs, including the left and right lungs, heart, esophagus, and spinal cord. Based on U-Net, Vesal *et al*. [90] used the expansion convolution and aggregation residual connection methods to segment OARs in chest CT images, and achieved high-precision segmentation of 20 undiscovered test samples.

GAN [61] can produce quite good output through mutual game learning of generative and discriminative models. Dong *et al*. [91] proposed a UNet-GAN strategy to automatically delineate the left and right lungs, spinal cord, esophagus, and heart. With the assistance of adversarial networks, the segmentation accuracy was greatly improved. It has been found in experiments that the traditional convolutional neural network model is not very compatible with medical imaging. He *et al*. [92] proposed a unified encoder–decoder architecture based on the U-Net model and used it in multi-task procedures. It is trained in learning mode, and the experimental results show that the DSC accuracy on the heart reaches 95%.

Zhao *et al*. [64] introduced multi-instance loss and conditional adversarial loss based on the FCN network to solve the segmentation problem under more severe pathological conditions, and the experiment obtained a DSC of 97.93%. Chen *et al*. [93] designed a weighted DSC based on the loss function of the coefficients is used to solve the problem of segmentation imbalance, and the experiment obtained a DSC of 97.55%.

The biggest challenge of DL in the medical field is the lack of annotated training sets. Hu *et al*. [94] used the Mask R-CNN architecture to combine supervised and unsupervised machine learning methods to automatically segment lungs on CT images and obtained the best results for lung segmentation. Research on automatic segmentation of OARs is not only important for radiotherapy but also provides inspiration and implications for other image segmentation algorithms.

Harten *et al*. [95] proposed various segmentation technologies based on different frameworks in combination with 2D-CNN and 3D-CNN to automatically segment four OARs: heart, aorta, trachea, and esophagus. The experimental results show that the best performance is achieved in DSC and HD. Akila *et al*. [96] proposed a convolutional deep wide network (CDWN) to segment lung regions in thoracic CT images. In the experiment, the DSC and ACC of the LIDC-IDRI dataset reached 95% and 98% respectively. Zhang, *et al*. [97] established a CNN network based on ResNet-101 for automatic segmentation of OARs, including lungs, esophagus, heart, liver, and spinal cord.

Table **5** details related work on OAR segmentation. Fig. (**6**) depicts the DSC accuracy for OAR segmentation in the searched literature, where the abscissa represents the reference numbers in Table **5** and the ordinate represents the DSC values, which are mostly above 0.8.

## 4 DISCUSSION

Although recent studies show that DSLC outperforms traditional segmentation methods in terms of efficiency and accuracy [100], it still faces some challenges.

### 4.1 Medical Imaging Problems

Tissues and organs in medical images have a high degree of similarity, especially in low-contrast images, where the segmentation target is very similar to the background and it is difficult to distinguish the boundaries. In terms of medicine, MRI images are preferable to CT as input because they provide better visualization [101]. In computer technology, new algorithms can be developed for solving the low-contrast problem of medical image segmentation. For example, 3D algorithmic networks should be used because they can adequately extract contextual spatial information from medical images compared to 2D networks, alleviating the problem of low contrast [102].

### 4.2 Dataset Size Issue

Obtaining medical images involves patient privacy issues, and the production of medical datasets requires professional doctors to label them. These two reasons lead to a scarcity of large medical datasets. However, training the model without a large number of samples hurts the robustness of the DL algorithm, resulting in overfitting of the trained model, and the small dataset cannot demonstrate the algorithm's generalization ability. These issues make the clinical application of DSLC more difficult. Moreover, apart from the datasets provided by some competitions with common standards, the datasets used by most researchers are of uneven quality, and the datasets created using specific scenarios to verify the overall performance of the algorithms are not convincing. In particular, most DSLC studies are based on single-point dataset training, which lacks diversity, and medical images in real situations have great differences due to race, age, gender, disease, *etc*., resulting in decreased model segmentation accuracy.

In light of the scarcity of medical datasets, various medical institutions could build large-scale datasets by sharing data in order to provide DL researchers with more expert annotated data under the premise of protecting patient privacy [103]. From the perspective of computer technology, DL researchers can also try to use transfer learning strategies [104] to pre-train network models as a way to alleviate the problem of limited data. Furthermore, medical image datasets can also be augmented by cropping, rotating, filling, and color-enhancing images through data augmentation methods.

### 4.3 Algorithmic Model Problems

The deeper the layers of the network model, the stronger the ability to extract features and the more complex the network structure. For the pixel-by-pixel classification task of lung images, expanding the number of layers of the network model is conducive to training a more accurate segmentation model. In addition, in order to extract and fuse multi-scale features of images, most researchers try to use more strategies for extracting features in the network, which undoubtedly increases the complexity of the network structure. As the number of network layers of the model increases, the ability to extract features, the data occupied by the GPU memory, and the time to train the model increase at the same time. Most algorithms reduce the training time by sacrificing a large amount of GPU space. This is not a long-term solution, and complex network structure has become a technical barrier limiting the improvement of model segmentation accuracy. It is worth considering how to strike a balance between network design, computing time, and cost. Hu *et al*. [94] used the improved Mask R-CNN architecture to achieve high-precision segmentation in DSC and combined it with the K-means method to improve the segmentation accuracy while reducing the model structure. At the same time, to avoid the constraints of GPU memory, we can try to use algorithms such as GAN to generate training data artificially to reduce the number of hidden layers or parameters of the network and to overcome hardware constraints to a certain extent.

### 4.4 Clinical Application Issues

The biggest difference between clinical medical applications and the experimental process is that there will be various unpredictable clinical situations [105]. If the DSLC only operates in a data environment similar to the training dataset, it will be difficult to respond correctly to emergencies. DSLC is required to be able to continuously learn to cope with clinical emergencies. In addition, DL algorithms also lack interpretability, it is difficult to fully understand which factors in the algorithm will lead to degraded segmentation performance, and it may not be possible to control the stability of OAR segmentation and GTV accuracy. If this uncertainty is used in clinical practice, it is very dangerous. Before DSLC is used clinically, relevant hospital personnel should conduct a thorough risk assessment, consider legal and ethical responsibilities, think about measures to deal with emergencies, and formulate a set of detailed standard procedures to protect the safety of patients. Computer-related researchers can also explore new network frameworks that enable models to learn experiences autonomously under unknown conditions, improve models' continuous learning ability, and reduce clinical application risks.

## CONCLUSION

In this paper, we investigated many kinds of studies, extracted common datasets and evaluation indicators for LTs, reviewed the basic theory of DL-related algorithms, and discussed and compared DSLC-related work from two aspects of LT and OAR segmentation. By improving the network framework and the segmentation accuracy, DSLC achieved satisfactory results in OAR segmentation of the lung and heart. However, it also has some challenges. To address these challenges, this paper presents an analysis and possible solutions. The author's knowledge is limited, and some important works may not be included in this paper. Hopefully, this review will deepen researchers’ understanding of lung cancer RT and DL, and stimulate collaboration between the two communities to develop a more specialized adjuvant lung cancer RT application system.

## Figures and Tables

**Fig. (1):**
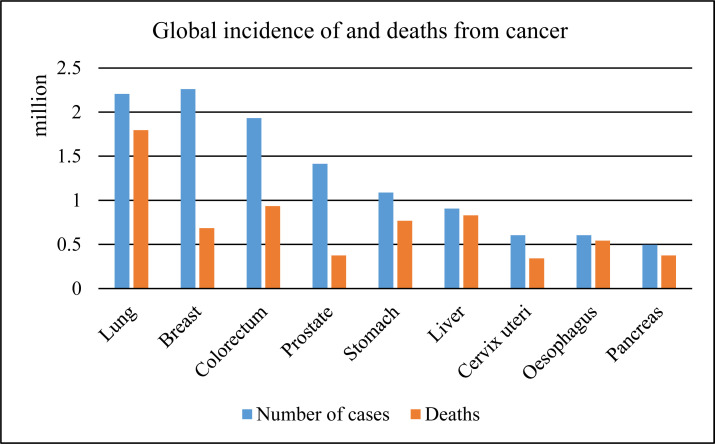
Global incidence of and deaths from cancer.

**Fig. (2):**
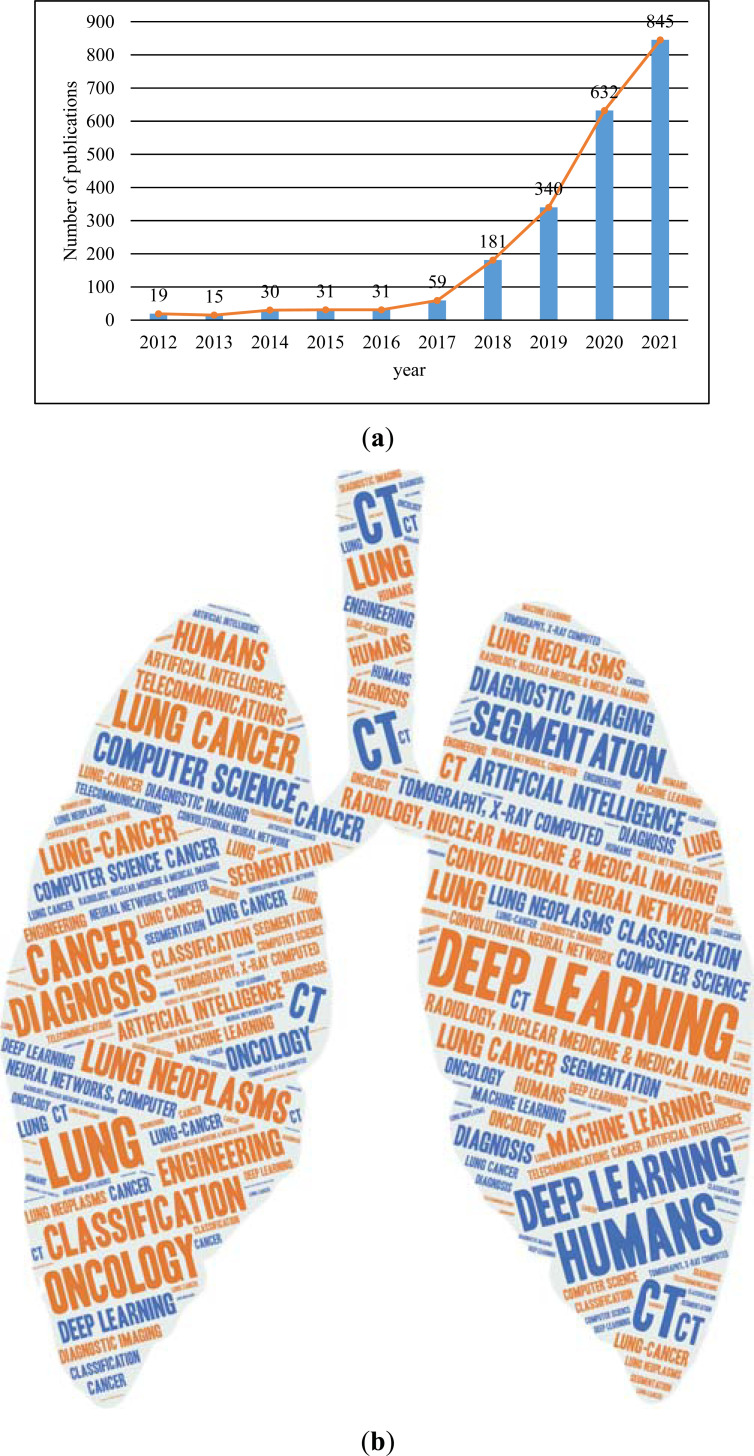
Literature research and analysis in the past 10 years. (**a**) Post trend chart; the x-axis represents the input year and the y-axis represents the number of posts. (**b**) Keyword analysis chart.

**Fig. (3):**
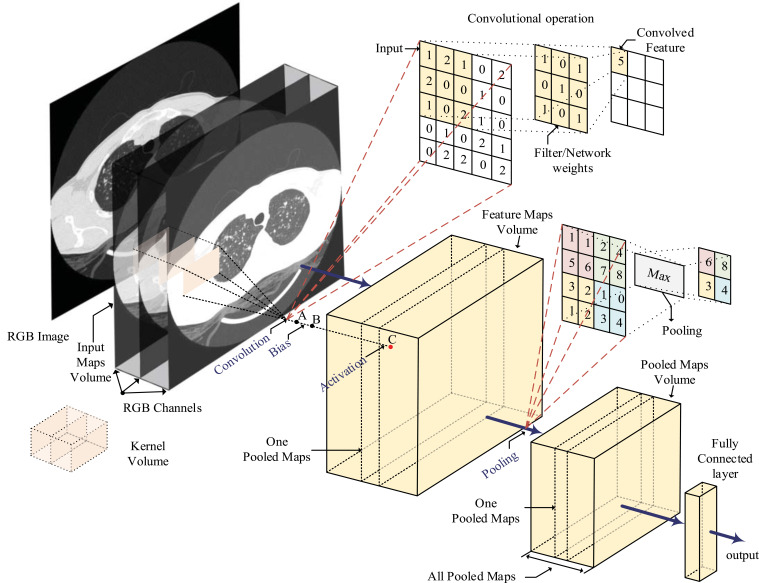
Convolutional neural network (CNN) structure diagram.

**Fig. (5):**
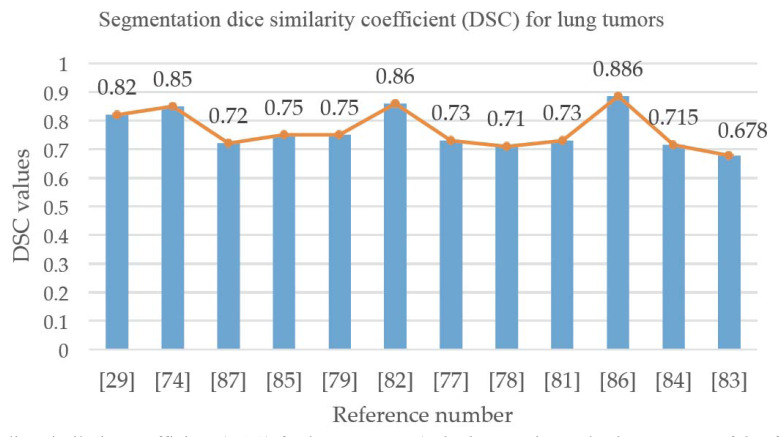
Segmentation dice similarity coefficient (DSC) for lung tumors.

**Fig. (6):**
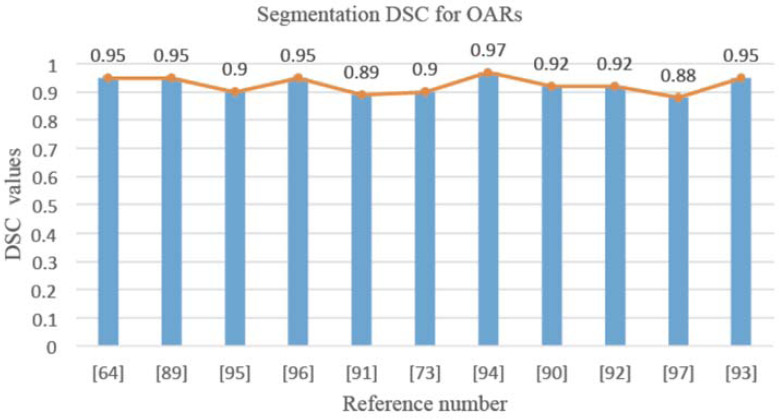
Segmentation DSC for OARs.

**Table 1:** Detailed analysis of our study compared to existing reviews.

**Refs.**	**Year**	**Literature Coverage** **Range**	**Type of Learning** **Methods**	**Main Theme**	**Existing ** **Surveys** **are Reviewed**	**Analyzed Research** **Trend**	**Metrics Details**	**Datasets Details**	**Current Challenge,** **Solutions**
[33]	2021	2013-2020	Deep Learning	Multi-organ segmentation	NO	YES	NO	NO	NO
[34]	2020	2017-2019	Traditional	Multi-organ tumor detection	NO	NO	NO	NO	NO
[35]	2019	2017-2019	Deep Learning	Lung cancer image analysis	NO	NO	NO	NO	NO
[36]	2018	2009-2018	Deep Learning and Traditional	Lung nodule detection	NO	NO	YES	NO	NO
[37]	2021	2017-2020	Deep Learning	Multi-organ segmentation and lung tumor segmentation	NO	NO	NO	NO	YES
[38]	2022	2013-2021	Deep Learning	Lung tumor segmentation	NO	NO	NO	NO	NO
OURS	2022	2018-2021	Deep Learning	Multi-organ segmentation and lung tumor segmentation	YES	YES	YES	YES	YES

**Table 2:** Public lung tumor datasets.

**Dataset**	**Year**	**Input**	**Details**	**Refs.**
Non-small cell lung cancer (NSCLC)	2021	CT/ PET-CT	285,411 images with a total data capacity of 97.6 GB	[39]
LIDC-IDRI	2020	CT/DX/CR	244,527 images with a total data capacity of 125 GB	[40]
Lung CT Segmentation Challenge 2017	2020	CT	9593 images with a total data capacity of 4.8 GB	[41]
NIH	2019	CT	32,735 images with a total data capacity of 221 GB	[42]
NLST	2017	CT	Over 75,000 CT images in 15 sub-databases	[43]
Data Science Bowl 2017	2017	CT	The National Cancer Institute’s Center for Cancer Research provides a two-stage dataset; the data capacity of the first stage exceeds 66 GB, and that of the second stage exceeds 38 GB	[44]
ChestX-ray14	2017	X-Ray	112,120 images with a total data capacity of 45 GB	[45]
QIN LUNG CT	2017	CT	3954 images with a total data capacity of 2.08 GB	[46]
LUNA16	2016	CT	888 CT images of 1084 tumors	[47]
SPIE-AAPM Lung CT Challenge	2016	CT	22,489 images with a total data capacity of 12.1 GB	[48]
LungCT-Diagnosis	2014	CT	4682 images with a total data capacity of 2.5 GB	[49]
TCIA	2021	MRI/CT	Large-scale public database containing medical images such as common tumors and corresponding clinical information, with all data organized and managed by TCIA.	[50]
TCGA	2021	MRI/CT	11,961 lung cases with a total data capacity of 2.5 PB	[51]
CLEF 2017	2017	N/A	CLEF dataset includes 500 patients, categorized into five TB types: invasive, focal, tubercular, miliary, and cavernous fibroma.	[52]
SCR	2000	X-Ray	247 chest X-rays, including left and right lung, left and right clavicle, heart, *etc*. Total data capacity of 2.5 MB	[53]
JSRT	2000	CT/X-Ray	154 conventional CT chest radiographs, the total data capacity of 1.33 GB	[54]

**Table 3:** Evaluation parameters.

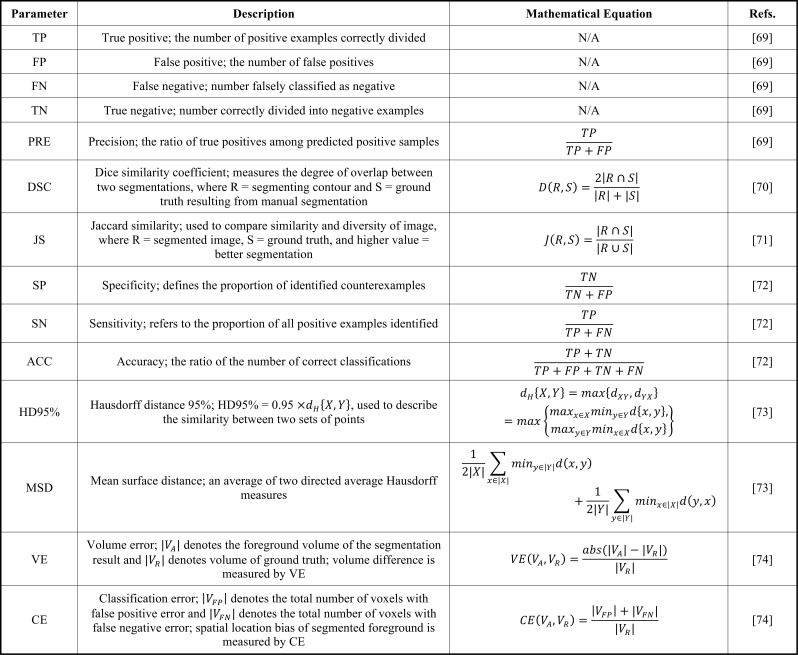

**Table 4:** Selected works on deep learning-based automated segmentation of lung tumors (LTs).

**Team**	**DataSets**	**Input**	**Net**	**Evaluation Metrics**	**Research Highlights**
Wang *et al*. (2018) [29]	9 patients	MRI	ANet	**DSC PRE SN** 0.82 ± 0.10 0.82± 0.08 0.85 ± 0.13	Adaptive neural network, A-Net, was introduced to delineate LTs
Zhao *et al*. (2018) [74]	84 patients	PET-CT	3D FCN	**DSC** 0.85 ± 0.08	Novel multimodal segmentation network, 3D FCN, proposed to integrate PET and CT images into the same utility
Jiang *et al*. (2018) [87]	1210 patients TCIA MSKCC LIDC	CT	MRRN	**DSC HD95% SN PRE** **TCIA** 0.74 7.94 0.80 0.73 **MSKC** 0.75 5.85 0.82 0.72 **LIDC** 0.68 2.60 0.85 0.67	Multi-resolution residual connection network proposed to combine features across multiple image resolution through residual connections to detect and segment LTs
Bi *et al*. (2019) [85]	269 patients	CT	ResNet	**DSC CV SDD** 0.75 ± 0.06 0.129±0.04 0.47±0.22	ResNet network used to segment LTs, obtaining better results than manual segmentation
Jiang J.*et al*. (2019) [79]	28 patients	MR -CT	U-Net + cross modality	**DSC VR HD95%** 0.75 ±0.12 0.19±0.15 9.36±6.00	Cross-modal deep learning (DL) segmentation method used to better segment LTs
Li *et al*. (2020) [82]	84 patients	PET-CT	3D FCN	**DSC VE CE** 0.86 ± 0.05 0.16±0.12 0.30±0.12	CT tumor probability maps and PET intensity images combined for accurate multimodal tumor segmentation
Zhang *et al*. (2020) [77]	330 patients	CT	ResNet	**DSC JS SN** 0.73±0.07 0.68±0.09 0.74±0.07	Fast segmentation of LTs using improved ResNet
Pang *et al*. (2020) [78]	NSCLC	CT	CTumor-GAN	**DSC PRE SN** 0.7108 0.7734 0.7042	Authors propose CTumorGAN algorithm for better segmentation of LTs
Leung *et al*. (2020) [81]	30 patients	PET	mU-Net	**DSC JS HD95%** 0.73 0.65 ±0.02 3.25±0.30	mU-N*et al*gorithm was used to segment smaller LTs on slices of PET
Jemaa *et al*. (2020) [86]	3664 trial scans	PET-CT	2D and 3D architecture	**Lymphoma Lung** **SN** 0.926 0.93 **DSC** 0.886 N/A	Combined with 2D and 3D convolutional networks, rapid detection and segmentation of LTs were realized
Fu *et al*. (2021) [84]	876 NSCLC 3063 soft tissue sarcoma (STS) [88]	PET-CT	MSAM	**NSCLC STS** **DSC** 0.7144 0.6226 **PRE** 0.7293 0.69 **SN** 0.8109 0.6494 **SP** 0.9995 0.9974	Based on attention mechanism, a multimodal spatial attention network module (MSAM) is proposed to strengthen learning of tumor-related
Lei *et al*. (2021) [83]	70 patients	PET-CT	RFN	**DSC** 0.6775±0.2341 **PRE** 0.7164±0.2728 **SN** 0.7318±0.2558	RFN network is proposed to segment LTs

**Table 5:** Selected works on deep learning-based automated segmentation of OARs for lung cancer.

**Team**	**DataSets**	**In**	**OARs**	**Network**	**DSC Metric**	**Other Evaluations Metric**	**Research ** **Highlights**
Zhao *et al*. (2018) [64]	LIDC-IDRI CLEF HUG [98]	CT	Lung	FCN	**LIDC**:0.9176 **CLEF**:0.9613 HUG**:0.9793**	**N/A**	Introduced multi-instance loss and conditional adversarial loss functions
Zhu *et al*. (2019) [89]	66 case of CT (30 case train, 36 case test)	CT	Lung Heart Esophagus Spinal cord liver	AdaptedU-Net	**Lung:**0.95±0.01 **Esophagus:**0.71±0.05 **Spinal cord:**0.79±0.03 **Heart:**0.91±0.03 Liver:**0.89±0.02**	**Lung:** 1.93±0.51(**MSD**) 7.96±2.57(**HD 95%**) **Esophagus:** 2.18±0.80(**MSD**) 7.83±2.85(**HD 95%**) **Spinal cord:** 1.25±0.23(**MSD**) 4.01±2.05(**HD 95%**) **Heart:** 2.92±1.51(**MSD**) 7.98±4.56(**HD 95%**) Liver:**3.21±0.93(** MSD**)**	Encoder–decoder U-Net neural network constructed based on convolutional neural networks (CNN) to automatically segment OARs
Harten *et al*. (2019) [95]	Seg Thor (60 thoracic CT scans)	CT	Heart Aorta Trachea esophagus	CNN	**Esophagus:**0.84±0.05 **Heart:**0.94±0.02 **Aorta:**0.93±0.01 Trachea:**0.91±0.02**	**HD** **Esophagus** 3.4±2.3 **Heart** 2.0±1.1 **Aorta** 2.7±3.6 Trachea **2.1±1.0**	2DCNN and 3DCNN frameworks combined to segment multiple organs at risk on chest CT
Akila *et al*. (2020) [96]	LIDC-IDRI	CT	Lung	CDWN	**0.95 ± 0.03**	**JS**:0.91 ± 0.04 **ACC**0.98 ± 0.01 **SP:**0.99±0.01 SN:**0.95 ±0.03** PRE:**0.95±0.03**	CDWN proposed for segmentation of lung regions in chest CT images
Dong *et al*. (2019) [91]	35 patiens	CT	Left lung Right lung Heart Esophagus Spinal cord	Unet-GAN	**Left Lung**0.97±0.01 **Right Lung**0.97±0.01 **Esophagus**0.75±0.08 **Spinal cord**0.90±0.04 Heart**0.87±0.05**	**Left Lung:** 0.61±0.73(**MSD**) 0.9989±0.0010(**SP**) 0.97±0.02**(SN)** 2.07±1.93(**HD 95%**) **Right Lung:** 0.65±0.53(**MSD**) 0.9992±0.0007(**SP**) 0.96±0.02(**SN**) 2.50±3.34(**HD 95%**) **Esophagus:** 1.05±0.66(**MSD**) 4.52±3.81(**HD 95%**) **Spinal cord:** 0.38±0.27(**MSD**) 1.19±0.46(**HD 95%**) **Heart:** **1.49±0.85(** MSD**) 4.58±3.67(**HD 95%**)**	U-Net used as generator and FCN as discriminator to design U-Net generative adversarial network (U-Net-GAN) to segment OARs in lung CT images
Feng *et al*. (2019) [73]	60 thoracic CT scans	CT	Left lung Right lung Heart Esophagus Spinal cord	3D U- Net	**Left Lung**0.98±0.01 **Right Lung**0.97±0.02 **Esophagus**0.73±0.09 **Spinal cord**0.89±0.04 Heart**0.93±0.02**	**Left Lung:** 0.59±0.29(**MSD**) 2.10±0.94(**HD 95%**) **Right Lung:** 0.93±0.57(**MSD**) 3.96±2.85(**HD 95%**) **Esophagus:** 2.34±2.38(**MSD**) 8.71±10.59(**HD 95%**) **Spinal cord:** 0.66±0.25(**MSD**) 1.89±0.63(**HD 95%**) **Heart:** **2.30±0.49(**MSD**) 6.57±1.50(**HD 95%**)**	Novel DCNN method proposed for automatic segmentation of chest OARs from large 3D images
Hu *et al*. (2020) [94]	1265 images	CT	Lung	Mask R-CNN	0.9733 ± 0.0324	**SN:**0.97 ± 0.09 **SP:**0.9711 ± 0.0365	Improved lung segmentation performance using a combination of Mask R-CNN and K-means
Vesal *et al*. (2019) [90]	60 patients	CT	Heart Aorta Trachea esophagus	2D Unet	**Esophagus:**0.858 **Heart** 0.941 **Aorta** 0.938 **Trachea**0.926	**HD** **Esophagus** 0.331 **Heart** 0.226 **Aorta** 0.297 **Trachea** 0.193	Introduced extended convolution in two-dimensional U-Net to better segment OARs
He *et al*. (2019) [92]	SegTHOR [99]	CT	Heart Aorta Trachea esophagus	Unet	**Esophagus:**0.8594 **Heart:**0.9500 **Aorta:** 0.9484 **Trachea:**0.9201	**HD** **Esophagus** 0.2743 **Heart** 0.1383 **Aorta** 0.1129 **Trachea** 0.1824	Optimized false positive filtering algorithm to decrease number of falsely segmented organ pixels
Zhang *et al*. (2020) [97]	250 patients	CT	Left lung Right lung Heart Esophagus Spinal cord liver	AS-CNN	**Left lung**0.948±0.013 **Left lung**0.943±0.015 **Esophagus**0.732±0.069 **Spinal cord**0.821±0.046 **Heart**0.893±0.048 **liver**0.937±0.027	**MSD:** **Left Lung**1.10±0.15 **Right Lung**2.23±2.33 **Esophagus**1.38±0.44 **Spinal cord**0.87±0.21 **Heart** 1.65±0.48 **Liver** 2.03±1.49	AS-CNN algorithm proposed, proving that DL is better than atlas method in automatic organ segmentation
Chen *et al*. (2019) [93]	45 thorax DECT	DECT	Left lung Right lung Liver Spleen Left Kidney Right Kidney	3D FCN	**L_lung**0.975±0.0064 **R_lung**0.976±0.0161 **Liver**0.962±0.0164 **Spleen:**0.914±0.0486 **L_Kidney:**0.937±0.0312 **R_Kidney:**0.945±0.0122	**HD** **Left Lung:** 6.97±2.67 **Right Lung:** 8.08±3.51 **Liver:** 9.64±4.89 **Spleen:**6.93±3.44 **Left Kidney**:4.41±2.17 **Right Kidney:**3.62±1.75	Multiple 3D CNNs proposed for segmentation of multi-organ DECT images

## LIST OF ABBREVIATIONS

ACC: Accuracy
CBCT: Cone-beam CT
CE: Classification Error
CNN: Convolutional Neural Networks
CT: Computed Tomography
CTV: Clinical Target Volume
DL: Deep Learning
DSC: Dice Similarity Coefficient
DSLC: Deep Learning-Based Segmentation Technology for Lung Cancer Radiotherapy
GAN: Generative Adversarial Network
GTV: Gross Tumor Volume
HD95%: Hausdorff_Distance95%
JS: Jaccard Similarity
LTs: Lung Tumors
MRI: Magnetic Resonance Imaging
MSAM: Multi-Modal Spatial Attention Network Module
MSD: The Mean Surface Distance
NSCLC: Non-Small Cell Lung Cancer
OARs: Organs at Risk
PET: Positron Emission Tomography
